# Review of Recent Metamaterial Microfluidic Sensors

**DOI:** 10.3390/s18010232

**Published:** 2018-01-15

**Authors:** Ahmed Salim, Sungjoon Lim

**Affiliations:** School of Electrical and Electronics Engineering, College of Engineering, Chung-Ang University, 221, Heukseok-Dong, Dongjak-Gu, Seoul 156-756, Korea; ahmedsalim789@gmail.com

**Keywords:** metamaterial, microfluidic, chemical sensor, biosensor, dielectric perturbation

## Abstract

Metamaterial elements/arrays exhibit a sensitive response to fluids yet with a small footprint, therefore, they have been an attractive choice to realize various sensing devices when integrated with microfluidic technology. Micro-channels made from inexpensive biocompatible materials avoid any contamination from environment and require only microliter–nanoliter sample for sensing. Simple design, easy fabrication process, light weight prototype, and instant measurements are advantages as compared to conventional (optical, electrochemical and biological) sensing systems. Inkjet-printed flexible sensors find their utilization in rapidly growing wearable electronics and health-monitoring flexible devices. Adequate sensitivity and repeatability of these low profile microfluidic sensors make them a potential candidate for point-of-care testing which novice patients can use reliably. Aside from degraded sensitivity and lack of selectivity in all practical microwave chemical sensors, they require an instrument, such as vector network analyzer for measurements and not readily available as a self-sustained portable sensor. This review article presents state-of-the-art metamaterial inspired microfluidic bio/chemical sensors (passive devices ranging from gigahertz to terahertz range) with an emphasis on metamaterial sensing circuit and microfluidic detection. We also highlight challenges and strategies to cope these issues which set future directions.

## 1. Introduction

Chemical sensors and bio-sensing devices have been developed for medical and environmental applications, explosive detection, and numerous industrial applications. In the next phase, researchers focused on implantable biosensors and detection of multiple analyte using a single sensing device. More importantly, biosensors have been contributed to non-invasive detection of malignant cells at initial stages and invention of drug discovery in medical research centers. State-of-the-art metamaterial (MM) inspired microfluidic bio and chemical sensors have been considered prominent among other electromagnetic sensors and are, therefore, selected for this comprehensive review. Starting from the fundamental principle of metamaterial structure, salient features of microfluidics sensors have been described. We also mention the challenges and strategies to mitigate them. Finally, we illustrate terahertz metamaterial based sensors to detect chemical and biomaterial.

Metamaterials are artificially defined periodic structures that simultaneously exhibit negative permittivity, and permeability [1,2]. By appropriate designing, they have been manipulated for various applications in the microwave and millimeterwave domain. The shape, geometry, orientation, and properly excited electric/magnetic field determine their behavior. For instance, a proper choice of metamaterials can determine whether the sensor is sensitive to electric or magnetic fields or both, and at what operating frequency the resonance should occur [3]. To refer to it as a metamaterial resonator which is designed from even a single unit cell conductive pattern coupled with microstrip line or coplanar waveguide (CPW) would be a misnomer because, using a single cell element, the criteria of periodicity and effective homogeneity cannot be satisfied in strict sense. The term “metamaterial based/inspired” seems to be an appropriate choice in these scenarios and we shall use this term throughout this review article. Metamaterial based circuits provide excellent performance and miniaturized footprints as compared to those based on substrate integrated waveguide (SIW) technology [4]. From design point of view, the electrical length and the characteristic impedance are important parameters [5].

**Figure 1:**
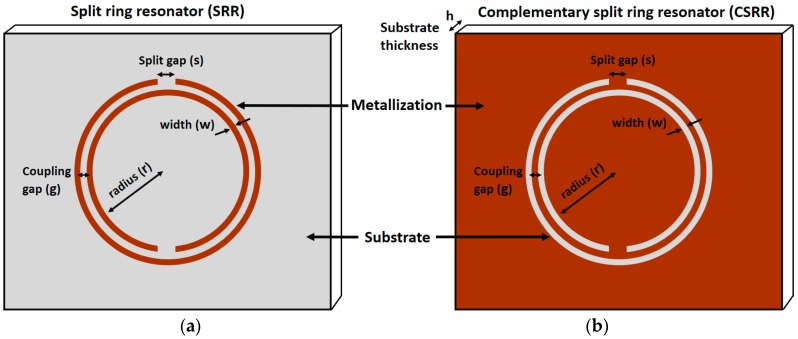
Metamaterial (unit cell) Topologies: (**a**) split ring resonator (SRR); and (**b**) complementary split ring resonator (CSRR).

Split ring resonators (SRR) and complementary split ring resonators (CSRR) are the two most exploited topologies of metamaterials, and have been utilized for synthesis of various structures/components, such as radio frequency (RF) Antennas [6,7], sensors [4], filters [8], and absorbers [9]. Various shapes of SRR and CSRR topologies have been investigated such as circular, square, triangular, etc. [10], and have been utilized depending upon applications’ requirements. SRR and CSRR are resonant elements and provide a high quality factor at microwave and millimeterwave frequencies [8]. SRR can be realized on the top/bottom surface of a substrate by rendering a ring shape pattern with an opening gap, as shown in Figure 1. The dimension (length/radius) of a unit cell metamaterial is much smaller than the wavelength of the external excitation (say, one tenth of a wavelength of excitation), which is why they can also be referred to as subwavelength structure. For instance, there have been proposed a CSRR based chemical sensor which resonates at λ/12 [11], and an SRR based biosensor which resonates at λ/15 [12]. CSRR is the dual of the SRR, and is realized by etching ring shape slots on top/bottom surface of the substrate. A time varying magnetic field is applied parallel to the ring axis and the current loops are generated in the rings [8]. The resonant frequency of the elements can be tuned by adjusting the device dimensions r, w, s, and g, which represent inner radius, width of ring/slot, split gap, and coupling gap between consecutive rings, respectively. Simulation tools help to identify the highest sensitive area of the structure. One possibility of highest electric field might be the split gap of SRR or along the slot(s) of CSRR which generates because of high capacitance and strong coupling. Using microfluidic tools, a foreign material (for instance, a dielectric, chemical, or biomolecule) is introduced onto the most sensitive area (the region showing the highest electric field magnitude). Here, the perturbation theory has been applied to alter the boundary conditions of the cavity/slot or other part of the structure. As a result, the effective dielectric constant changes and the resonant frequency increases/decreases. The amplitude of scattering parameters (S-parameters) also changes because of the losses. The higher is the difference in dielectric constants of the reference and the analyte, the wider is the shift in resonant frequency achieved.

Microfluidics is a rapidly emerging field that enables manipulation of extremely small (10^−9^ to 10^−18^ L) amounts of fluids using microchannels (dimensions of approximately 10–100 µm) [13]. The versatile applications of microfluidics have been contemplated to carry out analysis, screening, and detection of very small quantities of biomaterial and chemical samples. High resolution and sensitivity, low cost, rapid measurements, and small footprints for analytical devices are exquisite advantages of microfluidics. The capabilities of microfluidics are not yet limited to these, and have been proliferated even further. The concentration of some molecules in a sample can be controlled for a defined time and confined to a specific area [13]. The attractive characteristics of microfluidics stem from two salient features it offers, namely laminar flow and diffusive mixing [14]. The concise motivations behind the emergence of microfluidics have been witnessed in Whitesides’ article [13].

Computational fluid dynamics (CFD) has been widely applied to assist real-life applications by devising sophisticated and intelligent solutions. In this paragraph, we briefly mention some advanced applications inferred using CFD achievements, for instance, simulation-modeling of blood flow physiology and designing relevant devices [15]. During CFD simulations, defining an artery outlet boundary conditions for pulsatile flow, and accurately predicting the thrombotic cascade have been two non-trivial challenges [15]. They performed simulations and defined boundary conditions for blood flow arteries branch to/from vital organs of the abdominal and accounted for the effects of Hypertrophic cardiomyopathy (HCM) heart conditions [15]. Multi-objective optimization using various genetic algorithms have been investigated to enhance the efficiency and accuracy of simulations involved in designing a propeller, and ship [16,17]. Ship design optimization involves extensive testing and analysis of structure using complex iterative algorithms. To reduce the complexity of already existing particle swarm optimization (PSO) algorithms, an improved particle swarm optimization (IPSO) algorithm has been proposed in [17]. It improved the accuracy and efficiency of optimization, and the less-laborious modification in already existing design setup. Surrogate modeling is done to optimize the shape of fluidic oscillator (the results of which can be used to relate jet structure) [18]. To reduce computational cost without compromising over accuracy of optimized solution, grid independence test is carried out in CFD domain. In turbulence modeling velocity and pressure of a fluid flow are decomposed to averaged values [19]. In [20], three different turbulence models, such as k-ε, shear stress transport (SST), and baseline models were used and the k-ε model showed compliance with the experimental data [20]. The analytical expressions were developed using integrated fluidic flow across primary hydraulic fractures, stimulated reservoir volume (SRV) and unstimulated reservoir matrices.

Optical detection systems have been successfully served in the last few decades. Microscopy, dielectric characterization and analysis have been conducted using stains, fluorescent dyes and flow cytometry. They outperformed in terms of efficiency and specificity [21]. However, the equipment cost and being invasive for investigated cells were the major drawbacks [21]. Labeling technique in which fluorophores or preliminary are attached and may interact on the surface or induce modifications inside the cells [21]. In addition, cells-fixation and permeabilization required in the case of intracellular staining are time consuming and toxic for cells. Further processing on the same cells may not be possible due to contamination [21]. There was a drastic need to invent such a non-invasive biological analysis which could be performed without perturbation. Integration of microfluidics with microwave circuits emerged as an excellent technique for cells detection and bio-analysis. It has been established and gradually trying to oust the traditionally used approaches for bio/chemical analysis.

Few years ago, a radio frequency (RF) biosensor made from a planar resonant tank circuit detected up to 50 cells of HepaG2 (human cancer cell) albeit non-microfluidics [22]. A remarkable development was the convergence of microtechnology and microfluidics which augmented the microwave-based bio/chemical sensing. Recently, a single cell (for instance living B lymphoma cell) can be entrapped and detected, thanks to microwave-assisted dedicated microfluidic device [23]. This emerging detection mechanism has established several excellent features: (1) non-ionizing and contactless; (2) cells characterization directly in the biological medium; (3) without using any labeling; and (4) at the microscale (which fits the cells’ dimensions) [24]. Because of label-free and non-invasive characterization of biomaterials, cells can be kept alive during test and measurements [21]. In the meantime, biocompatible elastomers were also investigated. As a result, the real-time monitoring and cell manipulation have become accessible.

### Qualification Parameters of Microfluidic Sensors

The final goal of a sensor development is to launch it as a marketable product. To achieve this standard, a sensor should be simple, robust and easy to use [25]. Field applications (industry, military, security, and clinical laboratories) require portable sensors, while biomedical applications demand implantable sensors [25]. Miniaturization is an essential requirement in this scenario. Besides, it is important to reduce the sample (volume) required for testing and to mitigate the interferences [25]. Good durability of a sensor is achieved at the price of more sophisticated fabrication technology and utilizing expensive materials, which keeps a higher cost of product [25]. RF passive devices working as sensors require an initial calibration which needs to reinstate after each run. This calibration is not convenient in field applications; therefore, in certain cases, cheap and disposable sensors are designed for single use applications [25].

All chemical sensors are designed based on specific criteria. Sensitivity, hysteresis, stability, saturation, linearity, response time, and repeatability are characteristics to define the quality of a sensor [26]. Chemical sensors must be both sensitive and selective to a desired target specimen in a mixture of chemical samples. The calibration curve is an approach used to determine the concentration of analyte in an unknown specimen by comparing unknown to a set of reference samples having known concentration. The limit of detection (LOD) is also an important parameter associated with performance of chemical sensors. LOD is defined as the smallest concentration of an analyte that can be reliably detected, where reliable detection means the sensor response should be different from that of blank/reference [27]. Analytical LOD can be calculated as

LOD = 3.3 × (Standard Deviation of blank response/Slope of calibration curve)(1)

where the LOD is represented with the same units as that of concentration. For example, in [28], concentration of Fibroblast cells taken from a human male subject are detected in the range of 0 to 2000 cells/µL using a microfluidic SIW biosensor and the LOD is calculated as 213 cells/µL. Chemicals of various concentrations are loaded on the sensor/channels and sensor response is tuned. The electromagnetic sensors devised from resonators may have response/output in terms of their resonant frequency or equivalently S-parameters (magnitude). Most sensors respond to more than one type of chemical sample (having similar chemical structure as the target analyte), an undesired characteristic called cross sensitivity [29]. Multiple sensor sites on a chip having different selectivities can discriminate the un-correlated part of the signal relating to the concentration of an undesired substance [29]. Every practical sensor suffers some degree of interference from external noise or degradation of sensor response [25]. The complex permittivity of materials depends upon frequency and temperature, therefore, bio/chemists’ community demand establishing repeatability of measurements, especially for cells quantification [24].

## 2. Metamaterial Based Microfluidic Sensors

Cavity resonators have been a popular choice as sensing device owing to their high Q factor [30]. The Q factor of metamaterial (MM) based resonators is not as high as for cavity resonators, however, reduced resonator volume of MM structures is particularly attractive feature for chemical sensing. An increase sample filling factor within the cavity/gap/slot brings a strong interaction with electromagnetic field which corresponds to a higher sensitivity for dielectric measurements [30].

The key factor behind microwave bio/chemical sensors is that all biomaterials and commonly used solvents/chemicals have a uniquely identified complex permittivity in comparison with air and water. It is observed that there are some chemicals having exceptionally similar relative permittivity. For instance, the relative permittivity of Carbon tetrachloride (ε_r_ = 2.23) and Benzene (ε_r_ = 2.28) are identical to the first decimal digit [31]. However, there are very rare examples of chemicals exhibiting such minute difference of permittivity.

The fundamental principle of metamaterial based microfluidic microwave sensors depends upon dielectric perturbation phenomenon. It can be stated as: the electromagnetic boundary conditions are changed when a sample is intentionally introduced, as a result the resonant frequency and the quality factors are changed [32]. The change in resonant frequency is associated with a polarization of the material and the reduction in Q factor is associated with the dielectric loss of the material. Collaboratively, these have been used in analytical expressions to measure the complex relative permittivity of the sample, a well-known technique which researchers frequently use [30]. Generally, air (ε_r_ = 1) and water (ε_r_ = 80 @ 2.4 GHz) have been considered as the reference media.

### 2.1. Metamaterial Inspired Microfluidic Chemical Sensors Using Rigid Substrates

More than a decade ago, flame retardant (FR4) was widely used substrates for RF applications because of its low cost and still adequate performance. Currently, RT/Duroid (e.g., 5870, 5880, 6010.2 LM, and RO3000) have been the most utilized substrates to realize bio-chemical sensing devices, owing to its low loss properties and specially designed substrate for microwave and millimeterwave applications. Their stable dielectric properties (ε_r_ and tanδ) even at different temperatures and frequencies, make them a first choice for RF and microwave designs. In this section, we discuss metamaterial inspired microfluidic chemical sensors using rigid substrates.

**Figure 2:**
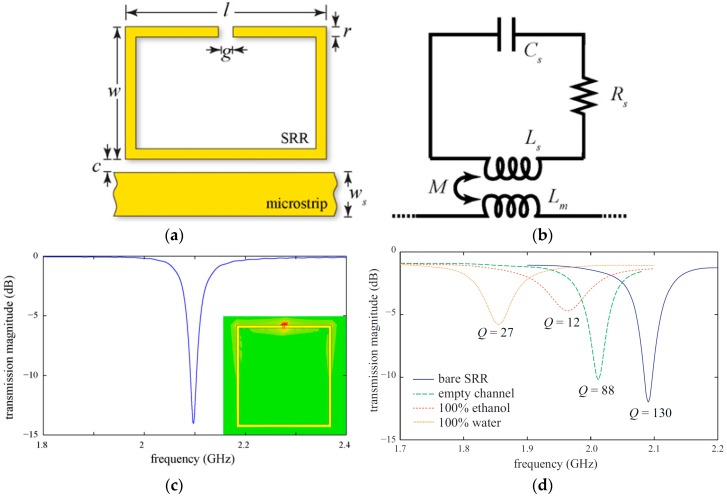

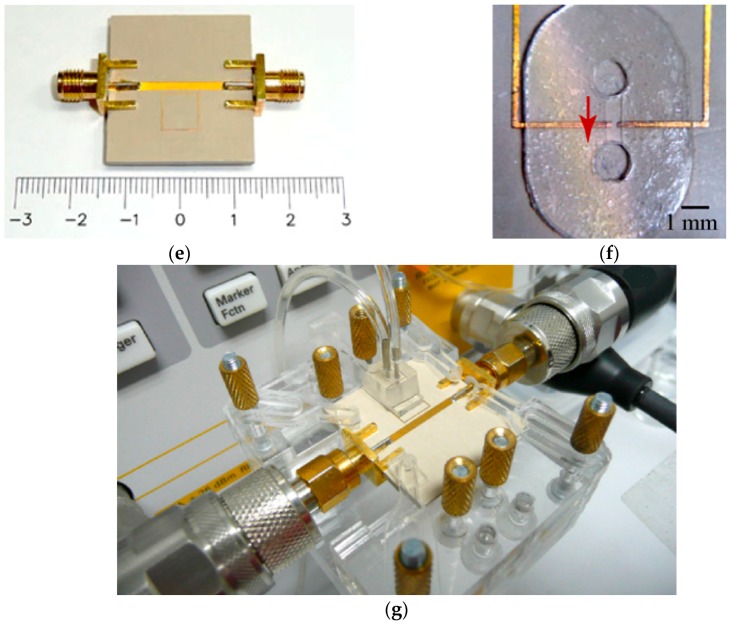
(**a**) Microstrip-coupled SRR etched on the top of a substrate; (**b**) Equivalent circuit of the proposed resonator with Lm represents the inductance of microstrip line, {RLC}s for the parasitic elements of the ring resonator, and M for the mutual inductance between SRR and microstrip line; (**c**) Simulated transmission magnitude of the proposed resonator, and in inset electric field magnitude at 2.1 GHz (resonant frequency) with the highest electric field at the split-gap of ring resonator; (**d**) Measured transmission magnitude showing various profiles of the proposed chemical sensor, 100% ethanol being the lossy chemical showing the lowest Q factor as compared to air and water; (**e**) Fabricated prototype assembled with two SMA (SubMiniature version A) connectors; (**f**) A zoom-in view showing the microfluidic channel accurately positioned on the SRR gap. The red arrow indicates the direction of fluidic flow; (**g**) Fully assembled sensor with polymer tubes delivering liquid into the chamber and coaxial cables connected to the vector network analyzer (Redrawn from [33]).

A microstrip coupled SRR on a RT/duroid 6010.2LM (Rogers Corporation, Connecticut, CT, USA) substrate (ε_r_ = 10.2, and a loss tangent of 0.0023) has been proposed as a chemical sensor, as shown in Figure 2 [33]. A polyethylene terephthalate (PET) film serving as microfluidic channel (made from craft-cutting), was positioned on the highest sensitive area of the ring resonator (the split-gap), to observe the maximum frequency shift. The complex permittivity of ethanol and methanol were characterized at 1.9 GHz, and the measurements were compared with the empirical values. The fabricated prototype sensor alone, and assembled in a complete sensing system is shown in Figure 2e,g.

A microstrip coupled CSRR has been proposed as a chemical sensor [34]. The microstrip line is designed on the top of substrate and CSRR is etched on the bottom ground. The concentration of water-ethanol solution was varied, and corresponding S-parameters were measured. To validate the proposed sensing system, the measured complex permittivity values of mixture were compared with the reference ones. This sensor showed four times higher sensitivity as compared to their previous work, as already described in [33].

A split ring cross resonator (SRCR) based absorber has been designed to propose as a microfluidic ethanol sensor [35]. The top layer is SRCR pattern on FR4, microfluidic channel is engraved in the middle layer FR4, and the bottom layer is one sided ground layer etched from FR4. For reflection coefficient measurements, two waveguide-rectangular (WR-90) adapters which are used for X-band (8–12 GHz) measurements, connected (using TE10 mode) to an Anritsu MS2038C vector network analyzer (Anritsu Corporation, Kanagawa Prefecture, Japan) were fixed on the top and bottom of the fabricated prototype. A shift of 1.04 GHz in resonance frequency has been observed for an ethanol concentration of 0% to 100%.

**Figure 3:**
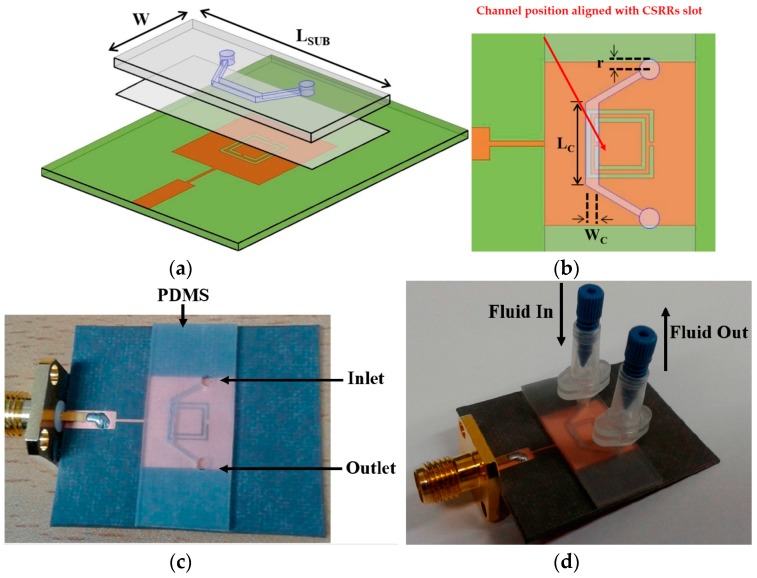
(**a**) Bird’s-eye view of the CSRR-loaded patch with an adhesive film and a microfluidic channel (top layer); (**b**) zoom-in view of channel alignment with CSRRs; (**c**) fabricated prototype of CSRR-loaded microfluidic patch as ethanol chemical sensor; and (**d**) side view with nanoport assembly (Redrawn from [4]).

Although our research group has already proposed a miniaturized chemical sensor using an eighth-mode SIW (EMSIW) antenna, despite its originality of work, it showed a limited sensitivity of 70 MHz shift in resonance frequency for liquid ethanol [36]. To enhance its sensitivity, metamaterial based design has been proposed. Metamaterial based structures provide high Q factor and small footprint, therefore, a CSRRs loaded patch has been utilized as an ethanol sensor [4]. It showed 270 MHz and 550 MHz shift in resonance frequencies with respect to DI-water and air, respectively. It was compact, non-invasive, and reusable. Two tightly coupled CSRRs are realized on a patch which has been fed by a combination of a quarterwave transformer and a microstrip line. A Polydimethylsiloxane (PDMS) based microfluidic channel etched using a laser cutting machine has been aligned at the highest sensitive area of CSRR slots (see Figure 3). An adhesive bonding film ARcare^®^92848 (Adhesives Research, Inc.) (Glen Rock, NJ, USA) has been used for bonding between substrate and PDMS layer. The detection of up to 10% ethanol concentrations have been reported.

**Figure 4:**
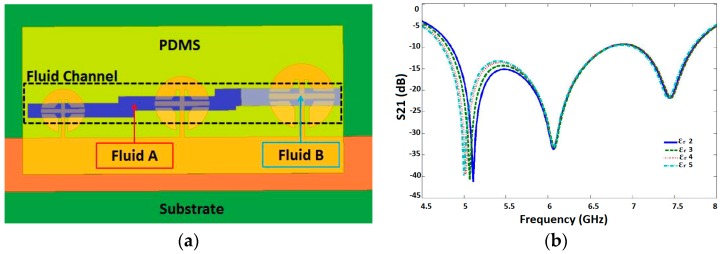
An OSRR coupled with microstrip line and integrated with a microfluidic channel proposed as a chemical sensor. The Spacing between OSRR elements with reference to outermost points (Dy) is optimized for all the proposed designs. (**a**) Simultaneous detection of multiple fluids, a pictorial view; and (**b**) insertion loss of the microfluidic sensor when permittivity of Fluid B was varied while keeping the permittivity of Fluid A constant (Redrawn from [37]).

An open split ring resonator (OSRR) coupled with a microfluidic channel has been proposed as a chemical sensor [37]. It has been designed on RO3003 substrate (h = 1.52 mm, ε_r_ = 3, tanδ = 0.0013) to operate at 6.5 GHz. The PDMS based microfluidic channels having 100 µm depth were developed and sealed with 50 µm thick polyimide film on backside of PDMS before placing on the sensor surface. They demonstrated measurements using a single unit-cell, an aperiodic array of three unit-cells, and an array of three different frequencies. To characterize the proposed sensor, various concentrations of Isopropanol, methanol, and glucose-d have been tested to measure the resonant frequency, Q factor and the phase constant of propagation. A remarkable feature of the proposed sensor was an attempt to investigate simultaneous detection of multiple fluids taking advantage of periodic structure, as shown in Figure 4a. The permittivity of Fluid A was kept constant (ε_r_ = 1), and the permittivity of Fluid B was varied (ε_r_ = 2, 3, 4, 5). Because each unit cell contributes the sensor response independently, the insertion loss around 5 GHz is switched for different permittivity of Fluid B, as can be seen in Figure 4b. However, variation in sensor response with variation in permittivity of both Fluid A and Fluid B could not be discussed.

To achieve high Q factor and compact design, a split-ring resonator with two gaps is proposed as a chemical sensor [30]. This double split-ring resonator (DSRR) is developed on RT/Duroid 5880 substrate, whereas microfluidic detection has been carried out using Quartz (ε_r_ = 3.8) and perfluoralkoxy (PFA) (ε_r_ = 2.1) tubes. Several commonly used chemicals were characterized, namely ethanol, methanol, chloroform, and hexane. The perturbation phenomenon has been applied by loading the microfluidic capillary onto the highest sensitive area of the DSRR. The measured resonant frequency, Q factor, and sensitivity were compared with simulation and calculation (using cavity perturbation theory), and found in good agreement for both Quartz and PFA tubes. As it was expected, the Hexane (ε_r_ = 1.8~2), and water (ε_r_ = 75~81 @ 2–5 GHz) showed the minimum (16 MHz) and maximum frequency shift (61.5 MHz) as compared to empty capillary (ε_r_ of air = 1). The reduction in the Q factor is associated to the higher loss tangent of the chemical. Therefore, water and ethanol exhibited, respectively, the highest and the lowest Q factor among the tested liquids. The measurements with PFA tube were relatively more accurate because of fixed position of capillary which certainly reduces the misalignment error.

**Figure 5:**
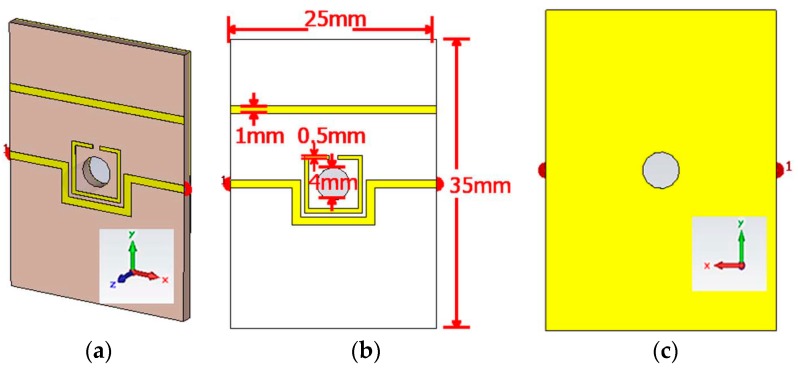
Metamaterial Based Microfluidic Sensor Design consists of a microstrip-coupled square shape ring resonator with one opening gap. A Microfluidic channel is positioned in the middle of the split ring resonator for a strong interaction of electric field with target analyte: (**a**) top view; (**b**) design dimensions; and (**c**) back view (redrawn from [38]).

A metamaterial based microfluidic chemical sensor is developed on RT/Duroid 5870 (h = 1.6 mm, ε_r_ = 2.33, and tangent loss = 0.0012) [38]. A U-shaped element for electromagnetic excitation and a patch is placed in front of the design as well as the Microfluidic gap, as shown in Figure 5. The unloaded resonant frequency was 4.62 GHz with a Q factor of 230. To validate the proposed chemical sensor, the complex permittivity of Ethanol, Methanol, Acetone and Poly Ethylene Glycol have been numerically and experimentally verified.

**Figure 6:**
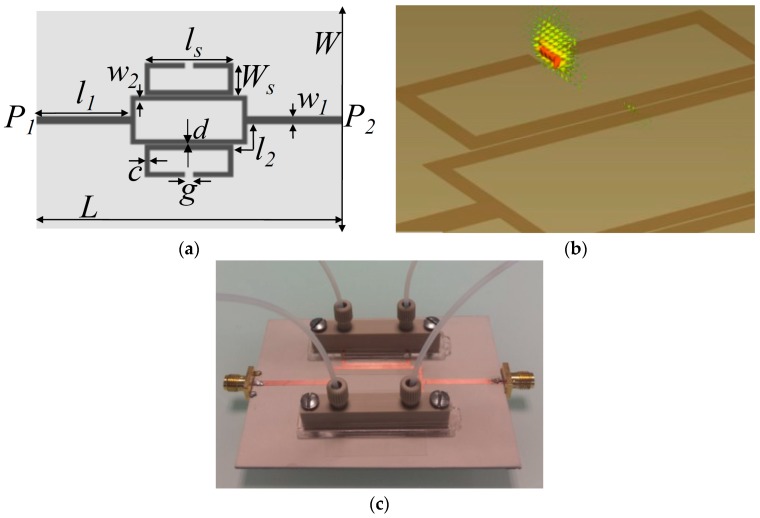
(**a**) Geometry of the splitter/combiner configuration: *L* = 86 mm, *W* = 62 mm, *l*_1_ = 27 mm, *w*_1_ = 2.22 mm, *ls* = 25 mm, *Ws* = 9 mm, *c* = 1.4 mm, *g* = 2.4 mm, *d* = 0.2 mm, *l*_2_ = 9.21 mm, *w*_2_ = 1.34 mm. (**b**) Electric field distribution at SRR resonance (the region with highest intensity at SRR gap is shown). (**c**) Fabricated prototype of metamaterial inspired microfluidic chemical sensor (redrawn from [39]).

Two SRRs coupled with microstrip line realized on a low-loss substrate (Rogers RO3010, ε_r_ = 10.2, tangent loss = 0.0023, and h = 1.27 mm) have been proposed as a chemical sensor [39]. By loading two asymmetric dielectric loads, two different resonant frequencies appear. Two microfluidic channels made from PDMS have been placed on the split gap of SRRs (i.e., region with the highest concentrated energy), as shown in Figure 6. To avoid a direct contact of injected-liquid with the sensor surface (substrate), which is necessary to prevent absorption, a thin layer (0.12 mm) of glass (ε_r_ = 5.5) has been placed between the PDMS layer and the Rogers substrate. The complex permittivity of mixtures of deionized water (DI-water) and ethanol were characterized.

**Figure 7:**
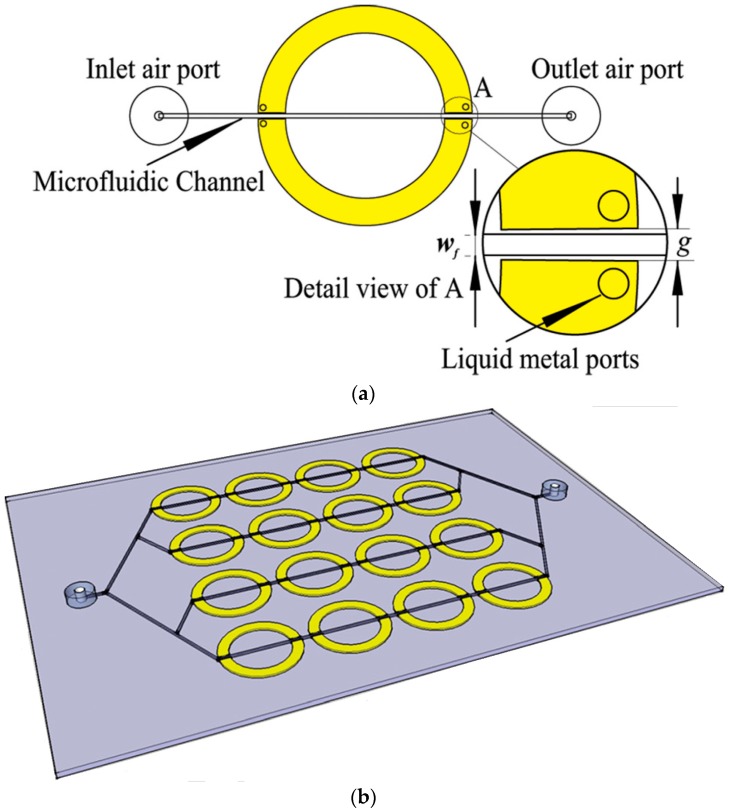
(**a**) Illustration of a unit cell meta-atom SRR based fully integrated microfluidic sensor with *g* = 0.8 mm, and *w_f_* = 0.4 mm; and (**b**) an integrated meta-surface SRR based sensor, consisting of 16 unit cells (redrawn from [40]).

The manual alignment of PDMS based channel (a common strategy in RF microfluidic sensors) onto the highest sensing area (mostly a narrow region) and its positioning may influence the measurement accuracy as well as sensor performance. Secondly, the adhesion/bonding of microfluidic channel to a non-microfluidic device as a separate layer cannot be considered as a fully integrated microfluidic sensor [40]. It is an inefficient approach when exploited as a stand-alone microfluidic sensor for lab-on-a-chip applications. In addition, these sensors require physical connectors (e.g., using SMA) for electrical connection with measurement setup (e.g., with VNA) to evaluate the sensing performance, making them inconvenient to use. To address these issues, an SRR meta-atom based fully integrated microfluidic sensor has been developed using PDMS for dielectric characterization of chemicals [40]. Instead of an additional external channel’s bonding to the sensitive area of SRR, an internal built-in channel is designed, passing through the split gaps of SRR (see Figure 7). The SRR micro-channels were photolithographically fabricated in PDMS, and then Galinstan is filled into the SRR microchannel, and the inlet and outlet of the microfluidic channel were sealed after the completion of injecting process. Finally, the flexible SRR (liquid metal encapsulated in PDMS) is peeled off from the silicon wafer. A 60 MHz shift in resonance frequency has been observed when water volume was increased from 10% to 90%. Comparatively tedious task of mold-cast PDMS and expensive Gallinstan may or may not be amiable for future researchers; nevertheless, it was an interesting approach.

Single channel per sensor for detection purpose is a limitation, most of the RF chemical sensors suffer with. In [41], a multichannel sensor array has been proposed for detection of multiple chemicals. Using a Rogers RT/Duroid 6010.2 LM (ε_r_ = 10.2, tanδ = 0.0023, and h = 1.9 mm), four SRRs having different dimensions were coupled with microstrip line and four distinguished resonances were achieved. By loading a 5 µL ethanol on split-gap of each SRR, four resonances were independently tuned. The absence of microfluidic channels makes the sensor non-usable for the next time, and, in addition, risk of contamination from pollutant particulates may occur.

**Figure 8:**
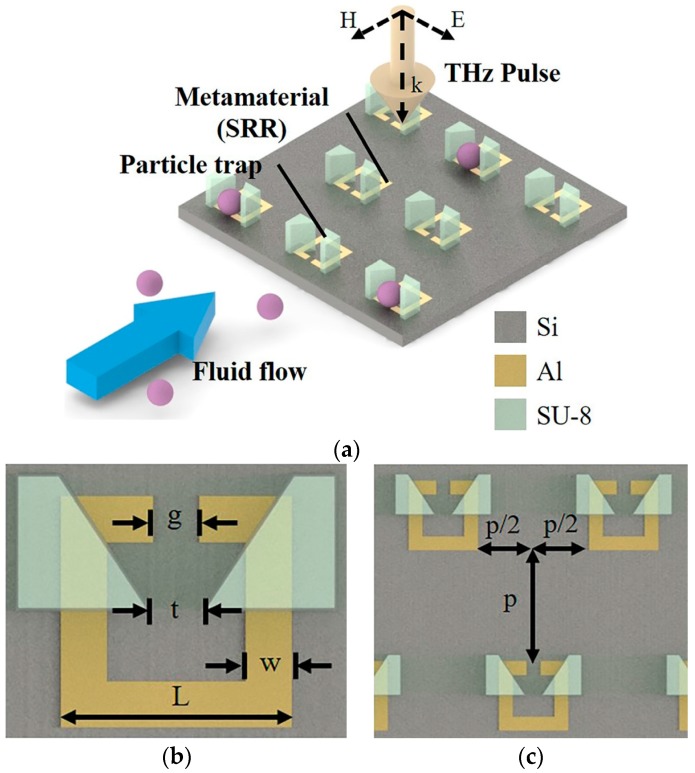
(**a**) SRR based sensor array (operating at THz frequency) integrated with a microfluidic system consisting of trapezoidal shaped structure to entrap the liquid particles of analyte. (**b**) Geometrical parameters of a unit cell (a SRR), and aligned-position of corresponding trapping structure, g = t = w = 5 µm, L = 30 µm; and (**c**) array design by integrating several unit cells from (**b**) with p = 50 µm (redrawn from [42]).

An SRR based sensor array (operating at THz frequency) has been integrated with a microfluidic system consisting of trapezoidal shaped structure to entrap the microparticles of analyte at the capacitive gap of SRR [42]. The increase in flow resistance between the two trapezoids after a particle is trapped, and the subsequent liquid is bypassed the trapped slot. This ensured the trapping of only one particle at the capacitive gap of each SRR (see Figure 8), which is critical for quantitative estimation of microparticles being trapped. They demonstrated the validity of the proposed design using polystyrene particles (each with a diameter of 20 µm) suspended in isopropyl alcohol solution. The maximum frequency shift of 10 GHz with a 15% particle trapping rate (observed from an optical microscope) has been achieved.

### 2.2. Metamaterial Inspired Microfluidic Chemical Sensors Using Flexible Substrates

Metamaterial based microfluidic sensors have shown great capabilities in dielectric based sensing. However, most of the fabrication approaches for these metamaterial-based sensors are complex, requiring complex and bulky equipment that must be operated in the cleanroom environment [3]. To address these concerns, metamaterial based microfluidic sensors have been proposed using flexible substrates such as paper, PDMS, polyimide, etc. Inkjet-printing, screen printing, and wax printing have been utilized to reduce the fabrication cost and complexity. However, using flexible substrates pose certain challenges: surface treatment, incompatibility with ink solutions (chemicals), and sensitive to thermal sintering to name a few. However, the great advantages of flexible substrates are value-added addition. They are low cost, easily available and most importantly compatible with additive manufacturing techniques. In this subsection, we discuss metamaterial inspired microfluidic chemical sensors which have been developed on flexible substrates.

**Figure 9:**
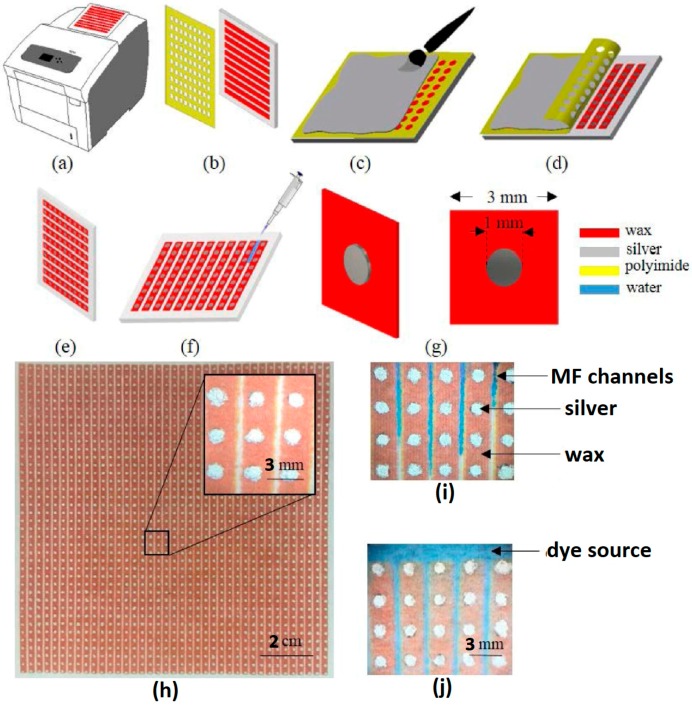
Fabrication process of metamaterial based microfluidic chemical sensor developed on paper substrate: (**a**) wax printer used for wax-printing on chromatography paper; (**b**) polyimide Sheet cut by CO_2_ laser used as interface layer on wax printed paper; (**c**) painting silver ink; (**d**) peeling polyimide sheet off the paper; (**e**) silver-painted paper after peeling off polyimide sheet; (**f**) flow of water through the microfluidic channels; (**g**) unit cell of the proposed sensor, the thickness of the chromatography paper and conductive silver disks are 90 μm and 100 μm, respectively; (**h**) fabricated sample (dimensions of 11.5 × 11.5 cm^2^), inhomogeneity of the silver disks due to low resolution in painting are shown on the inset figure; (**i**) flow of dye on microfluidic channel; and (**j**) channels are filled with dye, and the source of the dye is also shown (redrawn from [3]).

An array of disc-shaped resonator on chromatography paper (Whatman plc, Little Chalfont, Buckinghamshire, UK) using screen printing has been proposed [3]. As compared to sharp corner geometries, such as SRR, the disc-shaped is chosen because of its relaxed fabrication tolerance using screen printing. Moreover, a continuous track of microfluidic channel is easy to design rather than a complex 3D microfluidic structure in case of an SRR based sensor. To create microfluidic channels, wax patterning (ColorQube 8580, Xerox, Norwalk, CT, USA) has been utilized to make wax-printed areas hydrophobic and the rest hydrophilic. The complete fabrication process is shown in Figure 9. The sensor has been designed to operate at 94 GHz, and detection of oil (ε_r_ = 3.1), glycerol (ε_r_ = 57), methanol (ε_r_ = 33.1) and water (ε_r_ = 80.4) have been demonstrated.

**Figure 10:**
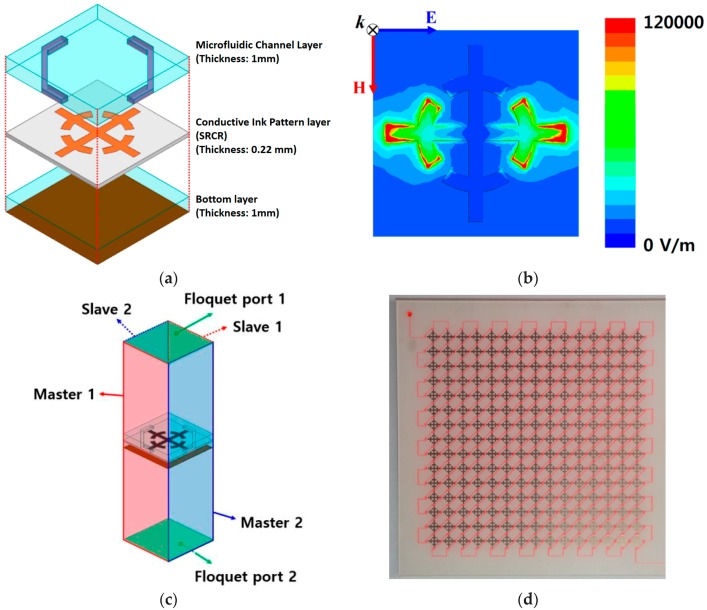
Flexible metasurface absorber is proposed as an ethanol sensor. SRCR is inkjet-printed on Kodak premium photo paper, serving as middle layer. Microfluidic channel is engraved on top layer (1 mm thick PDMS), and bottom PDMS layer is added to increase the absorbance. (**a**) Three-dimensional view of unit cell design; (**b**) electric field magnitude distribution of unit cell without loading a microfluidic channel; (**c**) simulation setup for boundary conditions and excitations; and (**d**) fabricated prototype filled with DI-water. Red ink is mixed with DI-water to make the fluidic path observable (redrawn from [48]).

Our research group has proposed several metamaterial inspired absorbers based on ingenious ideas [43,44,45,46,47]. They were targeted to either increase absorbance, or bandwidth, or angle insensitivity, or switching capability covering two different bands. However, these absorbers were based on rigid substrates. In [48], our research group proposed a metamaterial inspired flexible absorber which works as an ethanol sensor. Flexible devices are getting increasing attention because of their use in wearable applications, such as health monitoring as well as athletic training, and extensive use in upcoming technology including the internet of things (IoT) [49]. In parallel, inkjet printing on paper substrate provides attractive features, such as low cost and quick fabrication process [50]. In addition, it is environment friendly, hazardless toward user, and additive manufacturing technology (thus, cost effective) [51]. Split ring cross resonators (SRCRs) are inkjet-printed on a photo paper substrate, serving as middle layer of unit cell (see Figure 10) [48]. A microfluidic channel is engraved on top layer (1 mm thick PDMS), and bottom PDMS layer is added to increase the absorbance. The simulation modeling is done using HFSS (High frequency structure simulator) software. A unit cell geometry has been considered, where Master-Slave boundary conditions have been applied using floquet ports, such that the unit cell can be replicated along the two-dimensional direction. Because of periodic structure (repetitiveness of unit cell), floquet ports excitations are preferred over the wave ports. The sensor is tunable using microfluidic flow through microfluidic channel which is engraved inside a PDMS sheet, and up to 5% ethanol has been detected.

**Figure 11:**
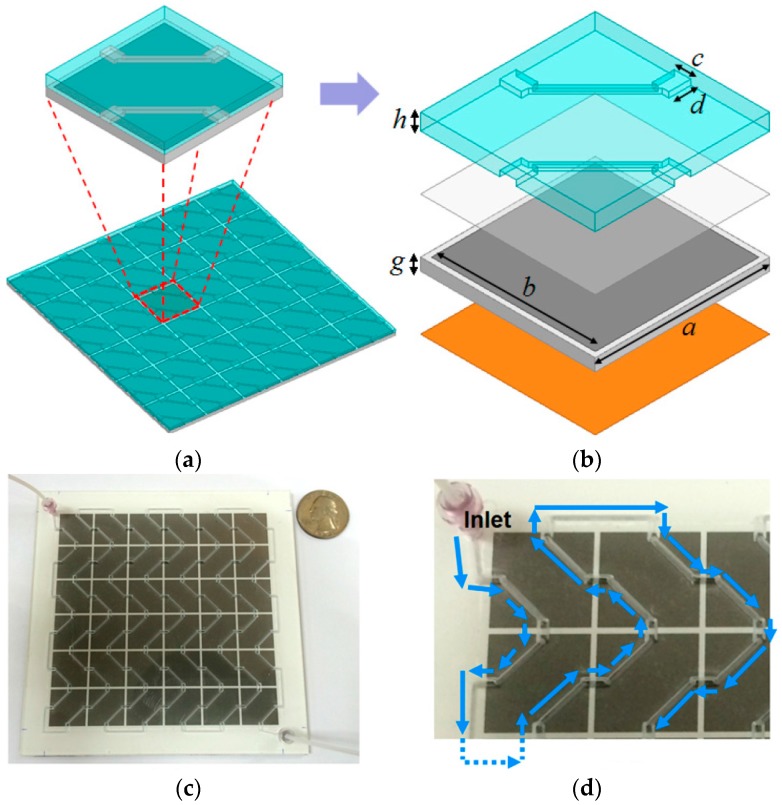
Microfluidic MM absorber as an ethanol sensor: (**a**) simulated model is shown; (**b**) unit cell consists of the microfluidic layer, bonding layer, and MM absorber layer with dimensions: *c* = 1.5 mm, *d* = 2 mm, *g* = 1.13 mm, and *h* = 1.5 mm; (**c**) top view of the fabricated prototype (overall structure in 6 × 6 array configuration with inlet/outlet tubes); and (**d**) fluid flow in the channel of the unit cell (blue color arrows) (redrawn from [9]).

A metamaterial inspired microfluidic absorber is proposed as a tunable ethanol sensor [9]. Considering a MM square patch as a unit cell, a periodic array consisting of several unit cells is inkjet-printed (Dimatix DMP-2831) on Kodak Premium Photo Paper (ε_r_ = 3.22, loss tangent = 0.09) (Office Depot, Atlanta, GA, USA) using ANP Silver-Jet 55LT-25C silver nanoparticle ink (Advanced Nano Products, Sejong, Korea). After considering the fabrication limits and enhanced, the microfluidic channels are engraved inside polymethyl methacrylate (PMMA with ε_r_ = 3.22, loss tangent = 0.02, and h = 1.5 mm) (McMaster-Carr, Atlanta, GA, USA) using laser etching machine. An adhesive SU-8 polymer layer (MicroChem Corp., Newton, MA, USA) is used to connect the top layer (microfluidic channel) with the bottom layer (conductive MM), as shown in Figure 11.

A split ring resonator with a T-shaped capacitive gap is proposed as microwave sensing device [52]. SRR has been coupled by an excitation loop fed by a coplanar transmission line. A PDMS (Sylgard 184, Dow Corning, Midland, MI, USA) has been used as a chip material. They demonstrated 330 MHz shift in resonance frequency for 3 nL water droplets.

To account for the losses introduced by materials (dielectric, and chemicals), accurate values of tangent loss must be used in simulation setup. The dielectric properties of commonly used materials to realize the microfluidic channel in bio-chemical sensors have been provided in Table 1.

To evaluate the performance of all the sensors discussed in this section, Table 2 is provided. Because the operating frequencies are very different, and the dielectric constant also depends on frequency, therefore a fractional variation in average frequency should be considered for a proper comparison. We introduce S_avg_, a performance metric in which frequency and dielectric constant tend to average out. S_avg_ represents percent change in average frequency shift per unit average ε and it is calculated as S_avg_ = (Δf/f_o_)/(Δε/ε_eff_) × 100%, where Δε = ε_water_ − ε_a_ with ε_a_ the dielectric constant of analyte, and ε_eff_ the variation in concentration of analyte. Its values are represented in percent.

### 2.3. Metamaterial Inspired Microfluidic Biosensors

In this section, we discuss and compare various techniques to detect biomaterials. We highlight the obvious drawbacks of conventionally used techniques for microorganism’s detection and radiation exposed detection systems. In these biosensing scenarios, we explain the significance of terahertz (THz) EM waves. We also briefly mention the metamaterial based non-microfluidic RF biosensors and their demerits as compared to metamaterial based microfluidic biosensors. In the end, we describe the plasmonic biosensors inspired from metamaterial structures and integrated with microfluidic tools.

In the 1980s–1990s, through several experiments, Schwan revealed the frequency dependent interaction of electromagnetic (EM) waves with biomaterial [59,60]. The complex permittivity and different polarization phenomena were found to be inter-related. The α, β, and γ dispersion corresponds to low frequency range, megahertz, and gigahertz regime, respectively. An enhanced interaction of EM waves and penetration into a single biological cell is possible only if high frequency EM waves are applied which are able to surpass the capacitive wall of cell membrane [21]. The γ dispersion provides such enriched information due to polarization of dipoles which occurs in the microwave and millimeterwave ranges. Another reason of this dispersion is reorientation dynamics of bio-molecules. Water is the largest constituent of living, and the dielectric relaxation by water molecules at/around 20 GHz is most well-known relaxation phenomenon. Therefore, dielectric properties of water at gigahertz regime essentially govern the dielectric properties of biomaterials, such as biological cells, tissue, proteins, skin, blood, and fat [32].

The detection of microorganisms has great interest because of its obvious applications in food industry and security systems. The polymerase chain reaction (PCR) and fluorescence-based systems have widely been used to detect microorganisms (bacteria, protozoa, algae, fungi, and viruses). Between these techniques, former is time consuming and labor intensive, while the latter requires fluorescent materials [61]. On the contrary, dyes are not required in optical scattering methods, for instance light scattering and auto fluorescence; however, they exhibit insufficient sensitivity [61]. There is a drastic need to envisage ingenious techniques for on-site detection of trace amounts of biological substances. The THz spectroscopy has enabled label-free, non-invasive, and sensitive detection using portable biosensors designed at terahertz range [61].

Mammography, computed tomography (CT), ultrasound, positron emission mammography (PEM), and magnetic resonance imaging (MRI) are various kinds of diagnostic radiology to detect early stage diseases [62]. They use low energy X-rays to collect very fine images of possibly affected area of human body. As per 2007, it has been estimated that more than 62 million CT scans per year are currently conducted in the United States, including at least 4 million for children [63]. No doubt, they are useful screening tools in diagnosis of diseases. However, increasing number of CT scans definitely expose more radiation doses which may induce cancer risks [63], even if we ignore the equipment cost, treatment cost and high-tech machinery involved in these screening systems. To complement these harmful effects, microwave imaging seems to be an option, where dielectric properties of healthy and malignant tissues can produce uniquely identified sensor-response [64]. Based on this approach, several RF microfluidic biosensors have been proposed and discussed in this review articles with an emphasis on those belong to metamaterial technology.

In 1998, Stuchly et al. developed probably the earliest form of modern RF biosensor [65]. Microwave biosensors composed of two main parts: the sensing element and a readout circuit [32]. The sensing part confines the EM waves and makes sure their interaction with the material under test. The bandwidth of sensing element classifies it as a narrowband sensor (resonator) or a broadband sensor. Narrowband sensors rely on resonator method which states that resonant frequency and quality factor of a dielectric resonator can be determined by its permittivity and permeability [32]. Usually, they are employed to determine low loss dielectric. It has been witnessed that academically proposed microwave biosensors are mostly based on dielectric perturbation phenomenon. Interaction of high frequency EM waves with biomaterials prevent electrode polarization which commonly occurs at low frequency.

In 2001, Facer et al. performed measurements of hemoglobin, *E. coli*, and DNA molecules in solutions using a two port coplanar waveguide (CPW) structure integrated with a microfluidic channel engraved in PDMS [66]. The measurements were performed from 40 Hz to 26.5 GHz without the need of surface functionalization and chemical binding. In 2008, Sanghyun et al. extracted permittivity of human embryonic kidney cells (HEK-293) in Dulbecco’s modified eagle medium (a popular cell culture medium) over a broad frequency range of 1–32 GHz [67]. A finite ground coplanar waveguide structure was developed on a pyrex 7740 glass substrate (550 µm thick) [68] and an SU-8 reservoir was realized to confine target biomaterial.

In 2008–2012, metamaterial based RF biosensors have been proposed using photolithography and MEMS technology [12,69,70,71]. They utilized the technique of antigen-antibody binding system directly on sensor surface (at the highest sensitive location of SRR), rather than utilizing microfluidic tools. They required surface treatments, such as immobilization of target molecules, additional coating of thin layers (gold, nickel) and masking layer to confine the binding molecules. In general, the sensitivity of these biosensors was reported in the range of 20 MHz to 150 MHz when target molecules alone and with binding pairs were introduced on the sensor surface. These non-microfluidic metamaterial inspired biosensors ([69,70,71]) require additional post-processing steps, such as surface treatment for functionalization and/or immobilization, and masking layer. Because of utilizing antigen-antibody binding system, they cannot retain non-contact feature. While describing the limitations of metamaterials based sensors, it has been reported that the performance of these structures is influenced to some extent, from the adsorption and desorption of analyte particles on the metamaterial surface [72]. Moreover, for verification purpose antigen-antibody binding step relies on optical detection system, for instance fluorescence microscope, which are expensive and bulky.

It is important to accurately plugin the properties of dielectric materials, and biological substances during simulation and modeling. More realistic values and simulation setup should be carefully handled for better system performance. To characterize the complex permittivity and conductivity of biological medium, using equivalent circuit models (obtained from inductance-capacitance (LC) resonators), analytical expressions have been developed [73,74]. Even though complex permittivity is a frequency dependent parameter, an approximate estimate is good to initiate the simulation. On the other hand, the value of loss tangent (tanδ) of biomolecules also changes with the cell-concentration. For instance, in [54], cancer cells (HepG2) have been characterized using an RF biosensor and they reported the average values of tanδ at different cell densities, such as 0.021 (2 × 10^1^ cells/μL), 0.032 (2 × 10^2^ cells/μL), 0.056 (1 × 10^3^ cells/μL) and 0.102 (2 × 10^3^ cells/μL). In Table 3, conductivity and permittivity of biological substances mentioned in various research studies have been given.

**Figure 12:**
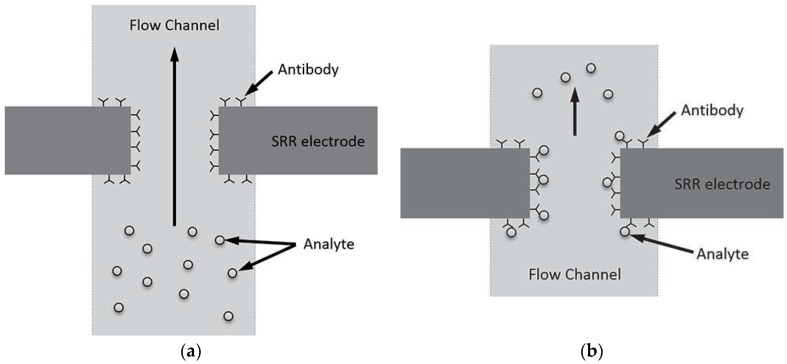
Antigen-antibody binding illustration: (**a**) before binding (with immobilized antibody around SRR electrode); and (**b**) after binding under analyte flow through a microfluidic channel (redrawn from [77]).

An SRR based label free biosensor integrated with microfluidics has been proposed to detect immunoglobulin G (IgG) [77]. SRR structure is realized on RT/duroid 60102.LM and microfluidic channel consists of PDMS which is formed by mold-casting technique. Anti IgG (Cystamine) has been used to immobilize antibody (IgG), which binds to IgG when a solution of (IgG + PBS) is injected in the channel. The illustration of binding system is shown in Figure 12. A shift of around 6.5–13 MHz has been observed in the resonance frequency of SRR (measured by S21) when the concentration of IgG is increased from 50 to 200 µg/mL.

Conventional techniques of optical detection rely on biomolecules to absorb light to generate fluorescence or direct absorption signals. If not all, most of the molecules are refractive, therefore, cannot be detected using conventional systems of optical detection [78]. Dielectric resonators can be considered as an option to detect non-absorbing molecules. However, extra efforts such as particular labeling and/or amplification are required to amplify the signal (resonance shift). Plasmonic biosensors for detection of single molecule have been proposed which do not require above mentioned extra efforts [78]. There have been developed several metamaterial based THz biosensor for enhanced sensitivity [79,80,81], and selectivity [82,83]. However, only those which have been integrated with microfluidic channel(s) are included in this review article.

Plasmonic metamaterial structures tend to generate a guided mode wave propagation (surface plasmonic polaritons (SPPs)) along the interface of metal-dielectric surface when incident light of a specific wavelength is exposed to structure. The distance between the nanorod metamaterials is smaller than the incident wavelength, thus they are called as subwavelength structure [84]. A change in position/intensity of plasmon absorption spectra takes place depending upon molecular adsorption [84], a property which is employed in nanoscale plasmonic biosensors.

**Figure 13:**
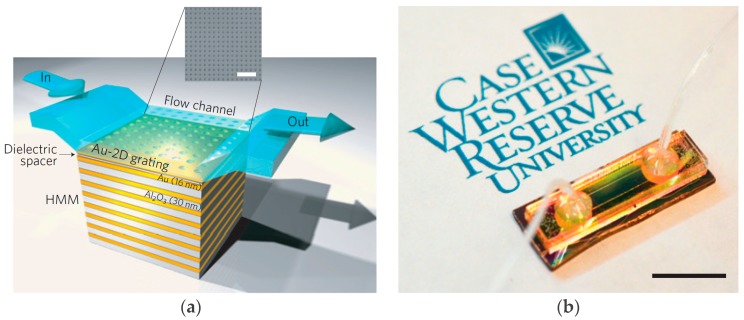

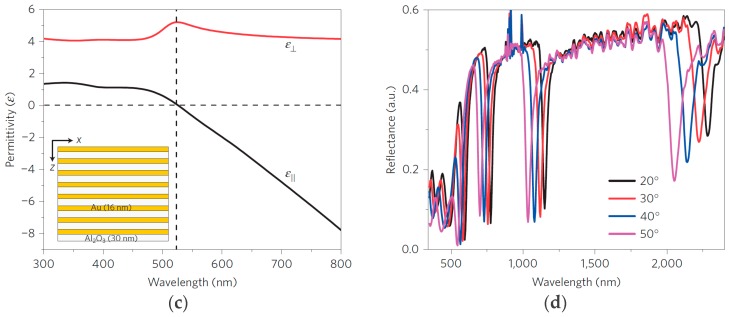
Hyperbolic metamaterial plasmonic biosensor integrated with microfluidics: (**a**) a 3D view of the fabricated biosensor with a fluid flow channel and a SEM image of the fabricated 2D subwavelength gold diffraction grating on top of the sensor surface; (**b**) photograph of the grating coupled hyperbolic metamaterial sensor device fully integrated with a microfluidic channel and sample tubing; (**c**) real parts of effective permittivity of biosensor, which shows a hyperbolic dispersion at λ ≥ 520 nm (dashed vertical line). In the inset the side view of fabricated biosensor is shown; and (**d**) reflectance spectra of the grating coupled HMM at different angles of incidence. It shows four prominent reflectance dips, corresponding to the bulk plasmon polariton modes, and two weak reflectance minima in the shorter wavelengths, corresponding to the SPP modes, all six modes are guided modes (redrawn from [85]).

A metamaterial based 2D nanorod gold arrays have been proposed as a plasmonic biosensor [84]. However, its enhanced sensitivity was specific to the infrared region, and the sensing mechanism was dependent on the bulk Kretschmann configuration. In addition, it is not suitable for point-of-care (POC) applications owing to the bulky, high-end instruments of the Kretschmann configuration [84]. To address these demerits, an extremely sensitive plasmonic biosensor has been proposed based on hyperbolic metamaterials [85]. Highly sensitive electric fields associated at visible and near infrared (NIR) wavelengths have been exploited to observe a change in the refractive index of its localized medium. Various biomolecules such as Glycerol-water, biotin-streptavidin binding, and bovine serum albumin solutions have been injected to evaluate the sensor response. Another remarkable feature of this biosensor was the detection of biomolecules having lower-molecular weight, for instance biotin with a molecular weight of 244 Da. The significance in this realm can be understood by knowing that previously reported plasmonic nanorod metamaterial sensors were able to detect biotin at concentrations as low as 10 µM while using a standard analytical technique. In [85], an enhanced sensitivity of up to six orders of magnitude (10 pM biotin in phosphate buffered saline) has been demonstrated. A 3D view of biosensor design and top view of fabricated prototype is shown in Figure 13.

**Figure 14:**
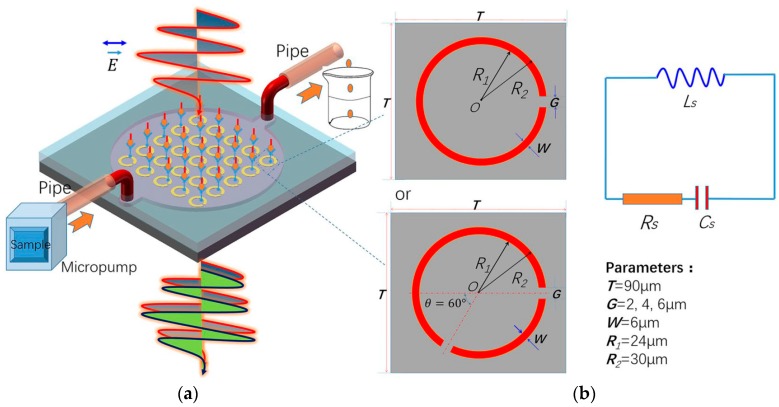
(**a**) THz metamaterials microfluidics biosensor; and (**b**) equivalent circuit with {RLC}s for the SRRs (redrawn from [86]).

The resonance frequency of Terahertz metamaterials becomes compatible with vibrational frequency of some biomolecules, for instance cancer biomarker [86]. To detect the Alpha fetoprotein (AFP) and Glutamine transferase isozymes II (GGT-II) of liver cancer biomarker in early stage, one gap SRR and two gap SRR based terahertz biosensors integrated with microfluidics have been proposed, as shown in Figure 14 [86]. The novelty of this design was to overcome the absorption of water into the trace biomolecules, an obstacle commonly arises at terahertz frequency. There were about 19 GHz resonance shift (5 mu/mL) and 14.2 GHz resonance shift (0.02524 μg/mL) for GGT-II and AFP with a two-gap-metamaterial, respectively.

## 3. Challenges and Strategies

Metamaterial inspired structures provide high Q factor (sharp resonance) and accomplish miniaturization. Since last decade, metamaterial inspired chemical sensors have been developed. Around 2010, several of these RF sensors did not utilize microfluidic technology. Because of absence of microfluidic channels, these sensors were not reusable and were unable to prevent contamination of particles from pollutant environment. Microfluidic tools integrated with RF sensors revolutionized the sensing technology. They require only microliter–nanoliter sample for testing, which is a matter of great interest for precious fluids, such as blood. Microfluidic channels are made from biocompatible material and sample under test remains safe. Advanced materials, coatings and adhesive films contributed to construct stable structure in which protection of substrate as well as bonding between microfluidic channel and substrate are satisfied. In general, the microfluidic channels are realized using low cost material and following simple fabrication process. All materials are of lossy nature, and, in that sense, they degrade the sensor performance to some extent, however, this drawback is not severe as compared to advantages of using microfluidic channels.

Most of the metamaterials inspired chemical sensors can detect only one analyte at a time because they use a single channel for sensing. To address this limitation, multichannel per sensor has been developed (e.g., [37,39,41]). Metamaterial inspired unit cells were collectively utilized to realize an array [37,39]. Dual/multichannel dielectric loading is used to tune corresponding resonant frequencies. In [39], simultaneous loading and detection of dual chemicals (using differential scheme) was carried out, although, one of the channel-loadings was fixed by a reference liquid. In [41], absence of microfluidic channel make the sensor vulnerable, as has already been discussed.

Microwave chemical sensors including those inspired from metamaterial structures suffer from lack of selectivity. Non-responsiveness toward undesired particles (for instance, interfering liquid in a sample matrix) is called as selectivity [87] and lack of it in microwave chemical sensors including those inspired from metamaterial structures is a serious issue. To address this concern, hybrid sensing has been proposed [88]. Hybrid sensors are based on microwave detection and they achieve selectivity using some chemical coating/layer. For instance, affinity of carbon nanotube toward ethanol is exploited in [88], where a thin layer of carbon nanotube is coated on a patch antenna to detect ethanol. In [89], a microwave gas sensor is built using hybrid technology. To detect ammonia gas, graphene sheets is coated on an SIW loaded ring slot/CSRR structure using chemical vapor deposition and a thin layer of polymethyl methacrylate (PMMA) is placed on top of graphene sheet. A change in conductance of graphene sheet (simulated sheet resistance set as 1000 Ω to 1550 Ω) occurs resulting from reception/donation of electrons when gas particles contact the graphene sheet. This electron exchange stimulates dielectric perturbation and it showed 59 MHz, and 157 MHz shift in measured resonance frequency, respectively.

Majority of microwave chemical sensors including those inspired from metamaterial elements are constructed using rigid substrates and they cannot be employed as wearable electronic devices. A growing trend in personal health monitoring devices and emerging technology, such as IoT, demand flexible, and bendable devices. Metamaterial inspired flexible chemical sensors have been proposed on photo paper using inkjet-printed technology [3,9,48].

Most of the electromagnetic chemical sensors including those based on microwave detection cannot work as an independent device/system for real time detection, unlike direct-reading instruments which are independent, portable devices. Metamaterial inspired chemical sensors (passive devices) remain non-functional unless they are connected to measuring instrument or processing equipment for instance, vector network analyzer (VNA). VNA is an expensive and bulky equipment. Moreover, it requires an initial calibration which is not affordable in the situations where portable sensors and instant measurements are required. Contrary to these passive sensing devices, biosensor incorporated with RF active systems can execute measurements using a simple and cost-effective instrument, such as digital multimeter [90].

Hyperbolic THz metamaterials based biosensor has been proposed exploiting plasmonic resonance [85]. In these kinds of biosensors, sensitivity was enhanced and they were made independent of bulky and high-tech machinery, drawbacks which existed in previous plasmonic biosensors [84]. It enabled the detection of biomolecules having lower-molecular weight (less than 500 Da), which are not possible to detect reliably with conventional plasmonic biosensors [84]. In [85], lower limit of detection (10 pM) was increased up to six orders of magnitude, making it an extremely sensitive biosensor. Metamaterial based biosensors sensitively detect chemicals and biomolecules, albeit their fabrication process (based on MEMS/photolithography) requires greater accuracy if compared to metamaterial based sensor designed at gigahertz range [72]. From fabrication point of view, metamaterials based biosensors have been suggested to improve sensitivity and accuracy [72]. Because of inability of conventional photolithography techniques to obtain high-resolution, and small feature sizes on unconventional substrates, a limitation which arises from photolithography [72]. Therefore, advanced fabrication technology must be established [72].

The electromagnetic sensors operating at gigahertz and terahertz regime mitigate challenges which otherwise arise when sensing is realized in the low frequency and megahertz range. High frequency electromagnetic waves interact with dielectric liquids (loaded in the microfluidic channel) causing a change in effective permittivity of dielectric substrate and surrounding region. The change in effective permittivity causes a shift in sensor response (either a resonance frequency or refractive index). The difficulty arises when a sensor responds to interfering liquids/particles (that may unintentionally be present with analyte and not desired to detect), an inherent drawback of RF sensors which rely on dielectric perturbation phenomenon. Therefore, new trends of bio-chemical sensing should shift towards hybrid sensing as a pragmatic approach, for instance RF sensor with additional coating/layer of chemical substance which would have affinity (specific) to target analyte. The occurrence of this chemical reactivity can be selectivity manipulated to target an analyte [87].

## 4. Conclusions

In this review article, we presented metamaterials based microfluidic sensors for detection of chemicals and biomolecules. These state-of-the-art microfluidic sensors are low cost, label free, and provide rapid detection with adequate sensitivity. The inherent nature of target analytes, i.e., lossy chemicals and biomolecules with low molecular weight, demand extremely sensitive sensing mechanisms in addition to sophisticated microfluidic tools. Advanced inkjet-printed techniques facilitate to realize novel sensors using elastomeric substrates, making these flexible sensors utilizable in wearable electronic devices.

## Figures and Tables

**Table 1:** Dielectric properties of commonly used materials to realize microfluidic channels in sensing devices.

Ref. #	Material	Permittivity ε_r_	Loss Tangent tanδ	Frequency [GHz]
[9]	PMMA (Poly methyl methacrylate)	3.2	0.02	3–4
[30]	PFA (perfluoralkoxy)	2.1	N/S	3
[30]	Quartz	3.8	N/S	3
[52]	PDMS	2.5–2.7	0.01–0.04	2–3
[53]	PTFE—Teflon (polytetra-fluoroethylene)	2.2	0.0002	N/S
[54]	Glass *	5.27	0.003	0–10

* (Corning EAGLE XG, Taiwan); N/S represents not-stated.

**Table 2:** Performance comparison of various metamaterial inspired microfluidic chemical sensors, presented in this review article.

Ref. #	f_o_ [GHz]	Δf [MHz]	ε_a_ *	ε_water_ **	S_avg_ [%]	MM Element	Analyte	Analyte Concentration	Flexible Substrate
[7]	3.5	270	5.08	73.77	7.51	CSRR	Ethanol	10‒90%	No
[9]	4	450	5.38	74.92	12.12	MM patch	Ethanol	0‒100%	Yes
[30] ^‡^	3	47.4	5.99	75	1.72	DSSR	Ethanol	0‒100%	No
[33]	1.9	110	8	76.5	6.49	SRR	Ethanol	0‒100%	No
[34]	2.4	400	6.6	76.6	16.58	CSRR	Ethanol	10‒90%	No
[35]	11	1040	5 ^†^	60	10.32	SRCR	Ethanol	0‒100%	No
[37]	6.5	575	10 ^††^	72	10.28	OSRR	Methanol	0‒100%	No
[38]	4.5	50	5.21	74.4	1.14	SRR	Ethanol	10‒95%	No
[39]	0.87	70	14	80	8.95	SRR	Ethanol	10‒90%	No
[40]	3	60	5.99	75	1.98	SRR	Ethanol	10‒90%	Yes
[48]	10	1140	5 ^†^	63.01	12.39	SRCR	Ethanol	0‒100%	Yes

* Real part of complex permittivity at different frequencies and at fixed temperature (20 °C) [55]; ** real part of complex permittivity of water at different frequencies and at fixed temperature (25 °C) [56]; ^‡^ exhibits a maximum of 61.5 MHz shift in resonance frequency corresponding to water and empty channel [30]; ^†^ real part of complex permittivity of ethanol at/above 10 GHz [57]; ^††^ real part of complex permittivity of methanol at 6.5 GHz (20 °C) [58].

**Table 3:** The conductivity and permittivity of biological substances.

Ref. #	Biological Substances	Conductivity [S/m]	Permittivity ε_r_
[61]	Fungi (Neurospora sitophila or Aspergillus niger)	N/S *	8
[62]	Breast Phantom (20:80) @ 3 GHz	0.91	12.13
[62]	Breast Tumor @ 3 GHz	3.14	55.45
[74]	Fat	0.03–0.06	5.9–6.6
[74]	Liver	0.4–0.5	65–81
[74]	Wet Skin	0.47–0.55	60–72
[75]	SW-620 cell line (colorectal cancerous cell)	0.02	N/S *
[76]	U87 glial cells (cells in central nervous system)	0.1	42
[28]	MRC-5 cell line (Human Lungs Fibroblast cells)	N/S *	53

* N/S represents not-stated; Note [62]: A significant difference in conductivity and permittivity of breast phantom and breast tumor provides a rich information to distinguish between healthy and malignant cells.
